# Synthesis of Novel Fluorescent Carbon Quantum Dots From *Rosa roxburghii* for Rapid and Highly Selective Detection of o-nitrophenol and Cellular Imaging

**DOI:** 10.3389/fchem.2020.00665

**Published:** 2020-07-31

**Authors:** Qianchun Zhang, Junyi Liang, Li Zhao, Yan Wang, Yuguo Zheng, Yun Wu, Li Jiang

**Affiliations:** School of Biology and Chemistry, Key Laboratory of Chemical Synthesis and Environmental Pollution Control-Remediation Technology of Guizhou Province, Xingyi Normal University for Nationalities, Xingyi, China

**Keywords:** carbon quantum dots, *Rosa roxburghii*, o-nitrophenol, Hep3B cells, cell imaging

## Abstract

A novel carbon quantum dots (CQDs) were successfully synthesized by one-step hydrothermal reaction using *Rosa roxburghii* as a biomass-based precursor. The CQDs have an average size of 2.5 nm and a narrow size distribution. They display strong blue fluorescence with a quantum yield of 24.8% and good biocompatibility. Notably, these CQDs were capable of detecting trace o-nitrophenol in surface water and sewage with high sensitivity and specificity. The linear range is 0.08–40 μmol/L, and the limit of detection is 15.2 nmol/L. Furthermore, this CQDs was successfully applied for o-nitrophenol analysis in river water and sewage samples. Additionally, Hep3B cells, a human hepatocellular carcinoma cell line, can be easily imaged with high resolution using the as-prepared CQDs as nanoprobes. These results reveal that the as-prepared CQDs have potential applications for detecting o-nitrophenol and cell imaging.

**Graphical Abstract:**
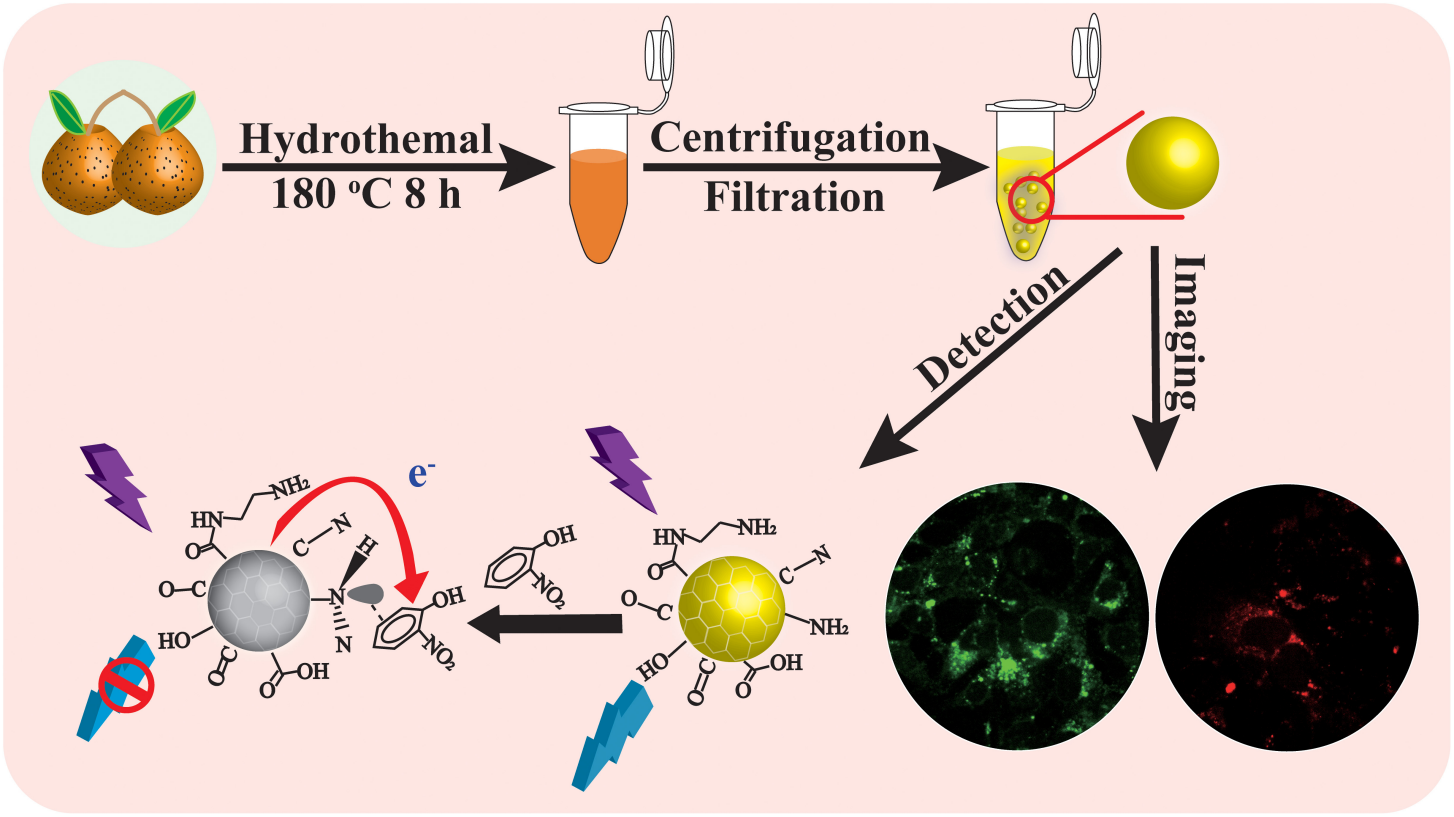
Scheme illustrates the synthesis of CQDs from *Rosa roxburghii* for detection of O-NP and cellular imaging.

## Introduction

As a new type of zero-dimensional carbon-based nanomaterials, fluorescent carbon quantum dots (CQDs) have received tremendous attention from the scientific community (Lim et al., 2015). Currently, two approaches are generally adopted to synthesize CQDs including top-down and bottom-up, both are using physical, chemical, or electrochemical techniques. These methods involve hydrothermal treatment, chemical oxidation, ultrasonic treatment, and microwave synthesis, etc., compared with other methods, hydrothermal treatment is convenient, sustainable, and powerful (Das et al., 2018; Jing et al., 2019). Hydrothermal carbonization of biomass is widely accepted and green method for the preparation of CQDs, among numerous precursors, natural biomass has been widely used as a carbon source for CQDs due to the low cost, eco-friendliness, low toxicity, and sustainability (Arul and Sethuraman, 2019). Examples include corn bract (Zhao et al., 2017), garlic cloves (Zhao et al., 2015), broccoli (Arumugam and Kim, 2018), aloe (Xu et al., 2015), quince fruit (Ramezani et al., 2018), cabbage (Alam et al., 2015), pumpkin (Gong et al., 2015), and others. However, CQDs derived from biomass general have low quantum yield (QY) (Das et al., 2018; Jing et al., 2019). Therefore, the precursors play a key role on improving the QY of CQDs. This limits their potential applications since CQDs with a high QY are suitable for a wide range of uses: fluorescent sensors (Yang et al., 2016), biosensing (Yan et al., 2019), chemical sensing (Yue et al., 2013; Lin et al., 2014), light emitting diodes (Wang et al., 2017), fluorescent ink (Atchudan et al., 2018), and optoelectronic devices (Yuan et al., 2019).

Nitrophenols in industrial wastewater cause pollution if discharged without proper treatment (Chatzimarkou et al., 2018). Thus, the analysis and monitoring of nitroaromatics in environmental samples are of great significance. In particular, o-nitrophenol (O-NP) is listed as a priority control substance due to its high potential to harm the environment and human health. Reported analytical methods include flow injection analysis (Junqueira et al., 2013), spectral analysis (Chen et al., 2019; Jiao et al., 2019), high-performance liquid chromatography (Zhao et al., 2018), electrochemical analysis (Yew et al., 2016), and capillary electrophoresis (Guo et al., 2004). However, most of these methods require sophisticated and costly instrument, and suffer from complex operations and low throughput in practical applications. In comparison, new methods using CQDs to detect organics are simpler, faster, cheaper, more sensitive and selective, and with a wider linear range (Qu et al., 2013). Therefore, it is desirable to develop a fast, sensitive, and selective method to detect O-NP using CQDs.

Another promising application of CQDs is bioimaging, considering their distinctive tunable color, low toxicity, good photostability, high biocompatibility, and resistance to photobleaching (Gong et al., 2016). The size of CQDs is usually <10 nm, and bioimaging requires a size smaller than 5 nm (Miao et al., 2017). Ding and coworkers established the feasibility of using full-color light-emitting carbon dots for *in vivo* imaging in mice and *in vitro* cell imaging, with a surface-state-controlled luminescence mechanism (Ding et al., 2016). Roy et al. reported the imaging of living human lung cancer cells (A549 and NCIH460) using CQDs (Roy et al., 2019).

In this work, a simple hydrothermal method was used to synthesize a novel highly fluorescent CQDs from *Rosa roxburghii* (sweet chestnut rose) as carbon source. The fruit part of *Rosa roxburghii* was chosen for the experiment because of its abundant output and low cost as a local natural wild fruit. Remarkably, fluorescence from the prepared CQDs could be selectively quenched by a low level of O-NP, and was used to measure O-NP in river water and sewage samples. The same CQDs also showed great potential in bioimaging, owing to their excellent biocompatibility and high fluorescence yield.

## Materials and Methods

### Materials

*Rosa roxburghii* was collected from the local farmers market and used fruit parts for subsequent experiments. Most of the chemicals used were obtained from Aladdin Chemistry Co., Ltd. (Shanghai, China): quinine sulfate, potassium chloride, lead chloride, barium nitrate, calcium hydroxide, sodium chloride, zinc chloride, anhydrous magnesium sulfate, chromium sesquioxide, copper chloride, nitrobenzene (C_6_H_5_NO_2_, NB), O-NP (C_6_H_5_NO_3_), 3-nitrotoluene (C_7_H_7_NO_2_, 3-NT), 3-nitrophenol (C_6_H_5_NO_3_, 3-NP), 2,4,6-trichlorophenol (C_6_H_3_Cl_3_O, 2,4,6-TCP), and 2,4-dinitrotoluene (C_7_H_6_N_2_O_4_, DNT). The following reagents were purchased from Kejin Biological Technology Reagent Co., Ltd. (Wuhan, China): 3-(4,5-dimethyl-2-thiazolyl)-2,5-diphenyl-2-H-tetrazolium bromide (MTT), phosphate buffered saline (PBS), fetal bovine serum (FBS), and dimethyl sulfoxide (DMSO). Mercuric chloride was obtained from Sinopharm Chemical Reagent Co., Ltd. (Shanghai, China). Penicillin-streptomycin was purchased from Solarbio Science and Technology Reagent Co., Ltd. (Beijing, China). The Hep3B cells were supplied by Chengdu Nuohe Biotech Co., Ltd. (Chengdu, China), as detailed cell authentication report in the Supporting Information. All reagents were of analytical grade and not further purified. The ultra-pure water was used in the experiments.

### Instrumentation and Characterization

Transmission electron microscopy (TEM) and high-resolution TEM (HRTEM) images of the CQDs were recorded on a Hitachi-F20 transmission electron microscope (Tokyo, Japan). The atomic force microscopy (AFM) images were recorded on a Bruker Multimode 8 AFM system (Karlsruhe, Germany). Photoluminescence (PL) measurements were conducted on a RF-6000 spectrofluorometer (Shimadzu, Japan). The X-ray photoelectron spectroscopy (XPS) analysis used an ESCALAB 250Xi (Thermo Fisher Scientific, Madison, USA) photoelectron spectrometer. Fourier transform infrared (FTIR) spectra were collected on a Bruker Nicolet 6700 spectrometer (Karlsruhe, Germany). UV-Vis absorption spectra were obtained on a 4501S UV-vis spectrophotometer (Gangdong, Tianjin, China) at room temperature. A KT7-900-434 high-speed centrifuge from Heller International Trading Co., Ltd. (Kenda, Germany) was used. The confocal microscopic images were acquired using a confocal laser scanning BX53 fluorescence–confocal microscope (Olympus, Japan). The pH value of the solution was measured by a S210 pH-meter (Mettler Toledo, Germany). All reagents were weighed with an AL104 electronic balance (Mettler Toledo Measurement Instrument Co, Ltd.; Shanghai, China).

### Synthesis of Carbon Quantum Dots

Firstly, *R. roxburghii* was washed, the calyx and stem were removed with stainless steel scissors, and the remaining flesh was cut into pieces and placed into a juicer. Next, 8.00 g of *R. roxburghii* pulp and 64 mL of water was added into a 100-mL Teflon lined autoclave, heated at 180°C for 8 h, and then cooled to room temperature. The brown solution was centrifuged at 12,000 r/min for 10 min and then further purified by a 0.22 μm filter membrane. The purified solution was stored at 4°C for further characterization and use.

### Determination of o-nitrophenol

In order to study the CQDs' response to various metal ions and organics, solutions of K^+^, Pb^2+^, Ba^2+^, Ca^2+^, Hg^2+^, Na^+^, Zn^2+^, Mg^2+^, Cr^3+^, Cu^2+^, 3-NT, 3-NP, DNT, 2,4,6-TCP, NB, and O-NP were mixed with CQDs, and the fluorescence was measured immediately after waiting for 30 min. Also, O-NP solutions were prepared at different concentrations. To each O-NP solution (0.50 mL), 4.50 mL of CQDs solution (125 mg/mL in 0.01 mol/L PBS, pH = 7.4) was added, and the mixture was kept for 30 min at room temperature. The fluorescence intensities before and after were recorded at 360 nm excitation. All measurements were repeated three times.

For analysis of O-NP in real samples, river water was collected from Dingxiao River (Xingyi, China), and sewage water was obtained from Jushan Sewage Treatment Plant (Xingyi, China). All original samples were pre-treated and centrifuged at 12,000 r/min for 10 min, and filtered through a 0.22 μm membrane to remove the colloidal particles. Then, 50 μL of the water sample was injected into the solution of CQDs, and the PL intensity was measured. The O-NP concentration in the environmental sample was determined by the calibration curve, which was obtained using pure O-NP solutions.

### MTT Assay and Cell Imaging

For cytotoxicity evaluation, Hep3B cells were incubated at 37°C for 24 h in Dulbecco's modified eagle medium (DMEM) with 1% penicillin/streptomycin, 10% heat-inactivated FBS. Subsequently, the cells were treated with different concentrations of CQDs (0, 0.10, 1.0, 10, 30, 50, 125, 250, 500, and 1,000 μg/mL) for 24 h. Next, 20 μL of MTT solution (5 mg/mL) was added to each well, followed by incubation for 4 h at 37°C in a dark, humidified environment. Afterwards, 150 μL of dimethyl sulfoxide (DMSO) was added to each well and shaken for 10 min. Finally, the absorbance values of the wells were measured using a microplate reader.

To evaluate the applicability of the CQDs as a practical fluorescent probe, *in vitro* imaging experiments were conducted. Hep3B cells were inoculated into 96-well plates using DMEM containing 10% FBS, in an environment with 5% CO_2_ at 37°C. Solutions with different concentrations of CQDs (0, 250, and 1,000 μg/mL) were added to each well and cultured for 24 h. Then, the precipitated cells were washed three times with PBS (0.01 mol/L, pH = 7.4). The stained cells were observed via laser confocal microscopy under green fluorescence (488 nm) and red fluorescence (561 nm).

## Results and Discussion

### Optimization of Synthesis Conditions

**Figure 1:**
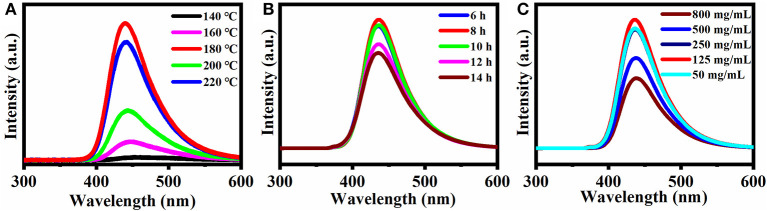
Effect of different conditions on the fluorescence spectra: **(A)** Synthesis temperature, **(B)** Synthesis reaction time, and **(C)** Concentration of CQDs in solution. Excitation wavelength: 360 nm.

Figure 1 summarizes the effects of synthesis conditions (reaction temperature, reaction time) and concentration of CQDs. All three factors strongly affect the fluorescence spectrum. In Figure 1A, the fluorescence intensity increased with increasing reaction temperature and reached a maximum at 180°C, which was chosen as the optimal reaction temperature for further experiments. In Figure 1B, the fluorescence intensity reached a maximum at a reaction time of 8 h. Therefore, 8 h was chosen as the optimal condition for the following experiments. Similarly, according to Figure 1C, the fluorescence intensity increased with the concentration of the CQDs from 50 to 125 mg/mL and reached a maximum at 125 mg/mL. At even higher concentrations, the fluorescence intensity decreased, possibly due to aggregation that caused fluorescence quenching. Therefore, 125 mg/mL was chosen as the optimal concentration for the preparation of the CQDs.

### Characterization of the Carbon Quantum Dots

#### Morphological Analysis

**Figure 2:**
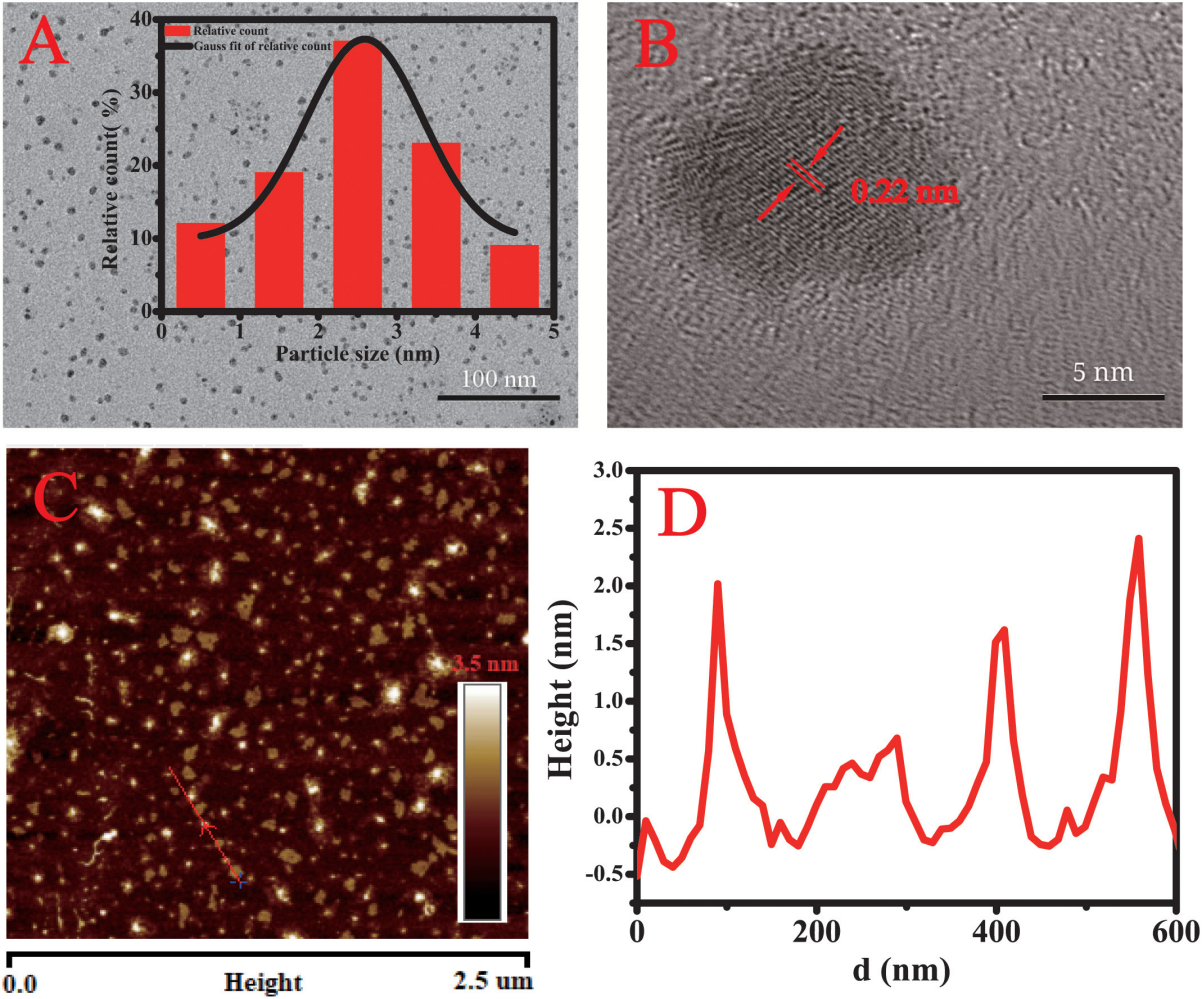
**(A)** TEM image of the CQDs and (inset) their size distribution. **(B)** HRTEM image of the CQDs. **(C)** AFM image showing the shapes of the CQDs. **(D)** Height profile along the line in the AFM image.

In the TEM image in Figure 2A, the CQDs displayed not only uniform size but also good dispersion. The size of individual CQDs was estimated from the TEM image by imageJ software and the distribution fitted to a Gaussian curve (inset). The average diameter was 2.5 nm, and the size ranged from 0.5 to 4.5 nm. The HRTEM image (Figure 2B) clearly exhibits lattice fringes with an interplanar spacing of 0.22 nm, corresponding to the (100) facets of graphitic carbon (Gao et al., 2018). In the AFM images (Figures 2C,D), the height of the CQDs particles is about 2.5 nm, which is consistent with the TEM results.

#### FTIR Analysis

**Figure 3:**
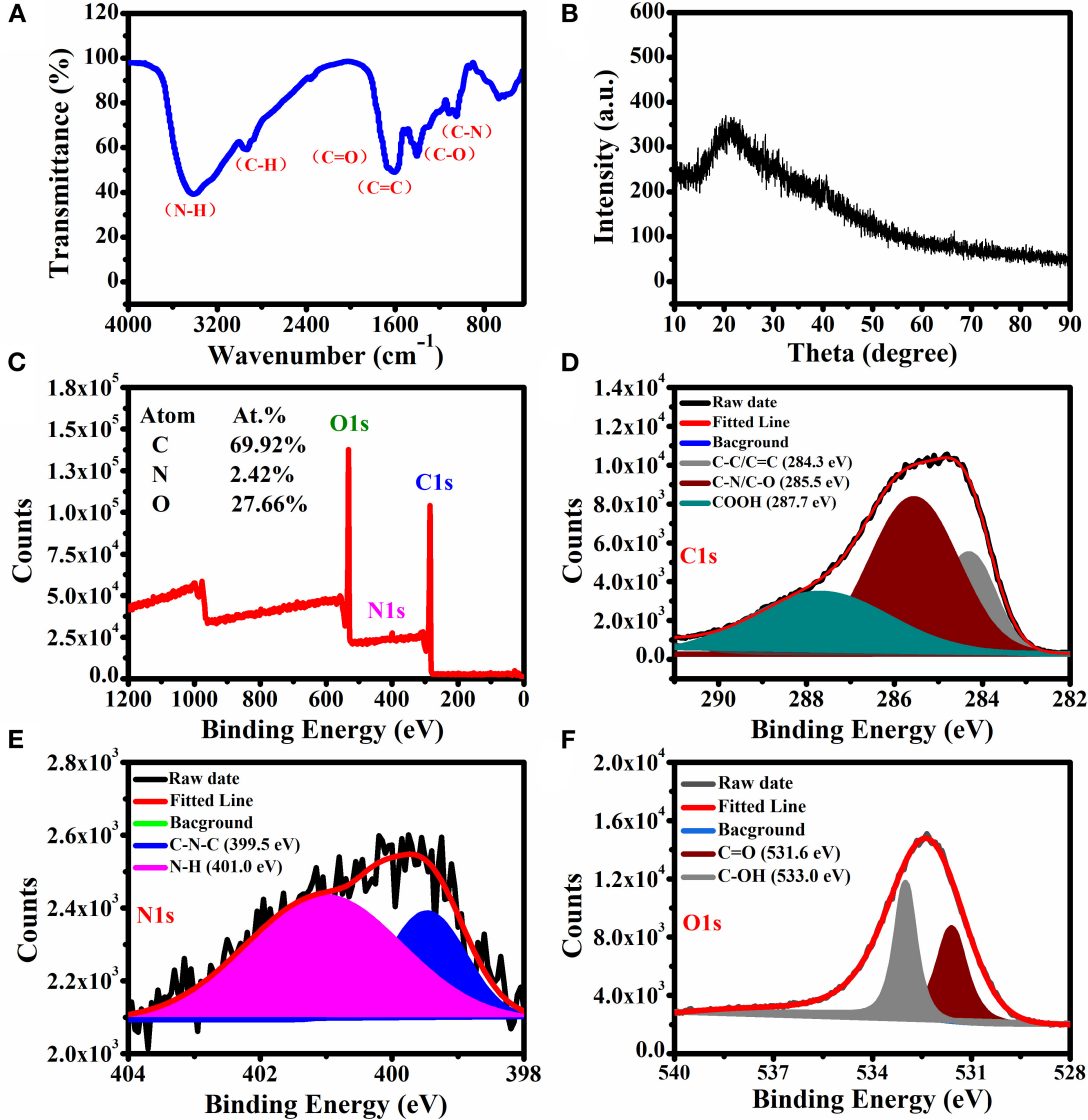
**(A)** FTIR spectrum, **(B)** XRD spectrum, and **(C)** XPS survey scan of the CQDs. High-resolution XPS spectra of **(D)** C1s, **(E)** N1s, and **(F)** O1s.

Functional groups on the surface of the CQDs were characterized by FTIR spectroscopy in the frequency range of 500–4,000 cm^−1^ (Figure 3A). In the spectrum, the characteristic peaks at 1,244, 1,637, and 1,703 cm^−1^ are assigned to the stretching frequencies of C–O, C=C, and C=O groups. A broad band was observed from 2,800 to 3,600 cm^−1^, demonstrating the presence of O–H/N–H bonds that contribute to the dispersion of nanocrystals in polar solvents (Huo et al., 2019). The absorption bands at 2,950 and 1,122 cm^−1^ could be assigned to the stretching frequencies of C–H and C–N bonds groups, respectively.

#### XRD Analysis

Figure 3B presents the XRD pattern for the synthesized CQDs. The sharp diffraction peak at 23.5° is consistent with the interlayer spacing of the graphitic structure (Da Silva Souza et al., 2018).

#### XPS Analysis

XPS was used to characterize the surface elements and their binding states. As shown in Figure 3C, the prepared CQDs exhibited C (69.92%), N (2.42%), and O (27.66%) elements on the surface, and the respective binding energies are 284.8, 399.9, and 532.6 eV. There are three peaks at 284.3, 285.5, and 287.7 eV, respectively, which were attributed to C–C/C=C, C–N/C–O, and –COOH groups in the high-resolution C1s spectrum (Figure 3D). Two peaks were observed in the high-resolution N1s spectrum (Figure 3E) and assigned to the groups of C–N–C (399.5 eV) and N–H (401.0 eV). In the O1s spectrum (Figure 3F), there are two peaks assigned to C=O and C–OH at 531.6 and 533.0 eV, respectively (Niu et al., 2015). These results are well consistent with those from FTIR. The identified surface hydrophilic groups provide the CQDs with good dispersibility in water.

### Photoluminescence Properties of the Carbon Quantum Dots

**Figure 4:**
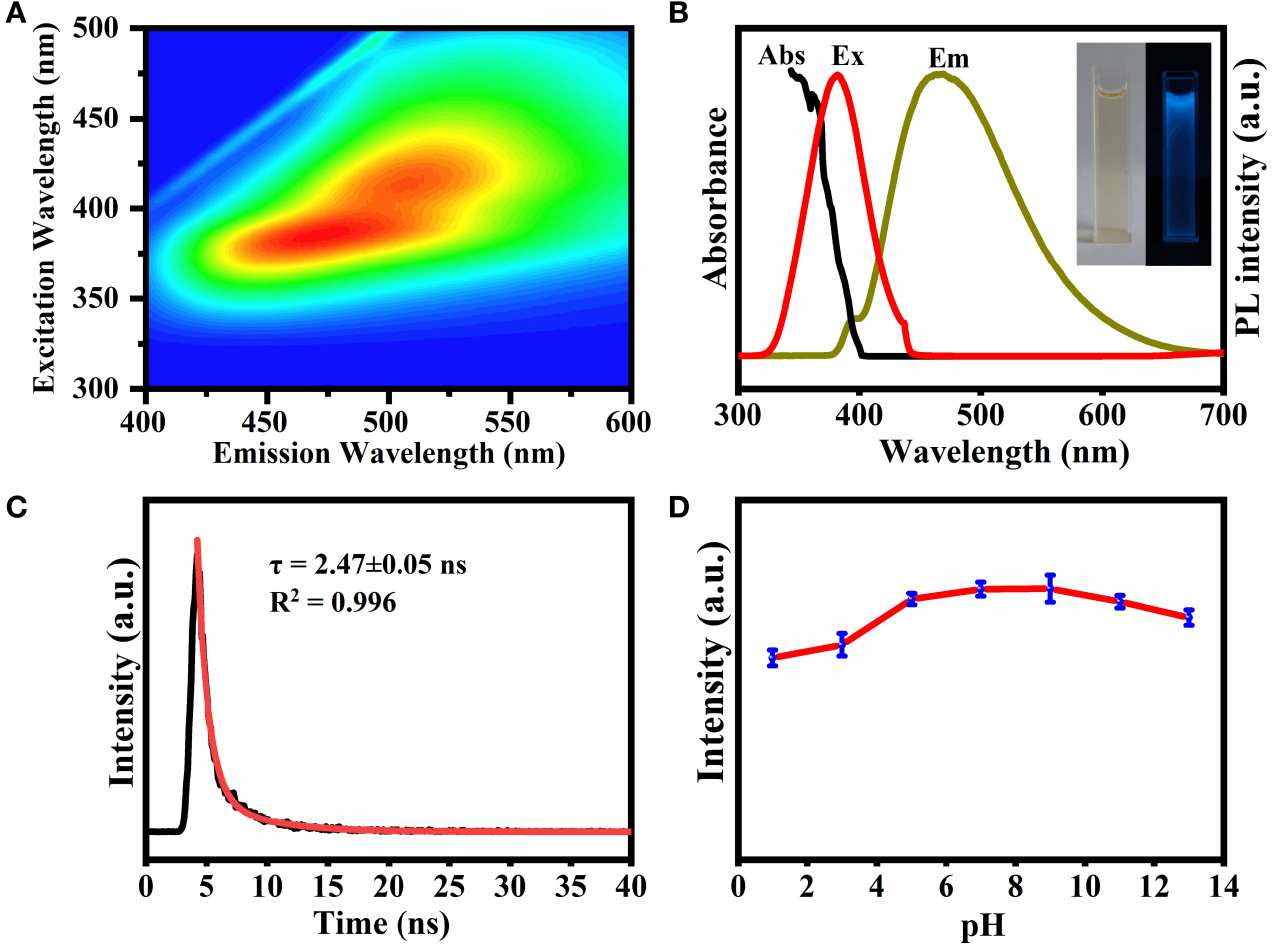
**(A)** Excitation-emission matrix for UV-vis absorption spectra of CQDs. **(B)** UV-vis absorption, PL excitation and emission spectra of the CQDs. Inset shows images of the CQDs under daylight (left) and UV irradiation (right). **(C)** Time-resolved PL decay for the CQDs. **(D)** Effect of pH on the fluorescence stability of CQDs. For the luminescence spectra, the excitation wavelength was kept at 360 nm.

Figure 4A shows the excitation-emission matrix of the prepared CQDs. When the excitation wavelength is in the range of 340–500 nm, the emission band is concentrated at about 400–600 nm depending on the excitation. When excited at 360 nm, the emission wavelength is 450 nm, indicating that the CQDs can convert near UV light to the visible range (Yang et al., 2017). The aqueous solution of CQDs (Figure 4B, left insert) exhibited strong blue PL (right) under UV irradiation (365 nm). The UV-vis absorption spectrum has a characteristic peak at 368 nm (Figure 4B), which can be assigned to the n–π^*^ transition of C=O in the as-prepared CQDs (Miao et al., 2017).

Additionally, the time-resolved PL decay curve in Figure 4C could be fitted well with a single-exponential function (lifetime: 2.47 ± 0.05 ns), implying that the PL is dominated by a single emission state. As revealed in Figure 4D, the fluorescence intensity increased with increasing pH from 1.0 to 9.0 and then decreased at higher pH. The reason may be that at first the ratio of protonated CQDs decreases with increasing pH, and then the deprotonation causes the net surface charge to decrease (Liu et al., 2018). The latter leads to partial aggregation of the CQDs and therefore a reduced fluorescence intensity. In the figure, the fluorescence intensity of CQDs did not decrease significantly within the pH range of 7.0–9.0, making this material suitable for bioimaging applications. The fluorescence QY of the CQDs in solution is about 24.8% at neutral pH.

### Fluorescent Probe for Detecting o-nitrophenol

**Figure 5:**
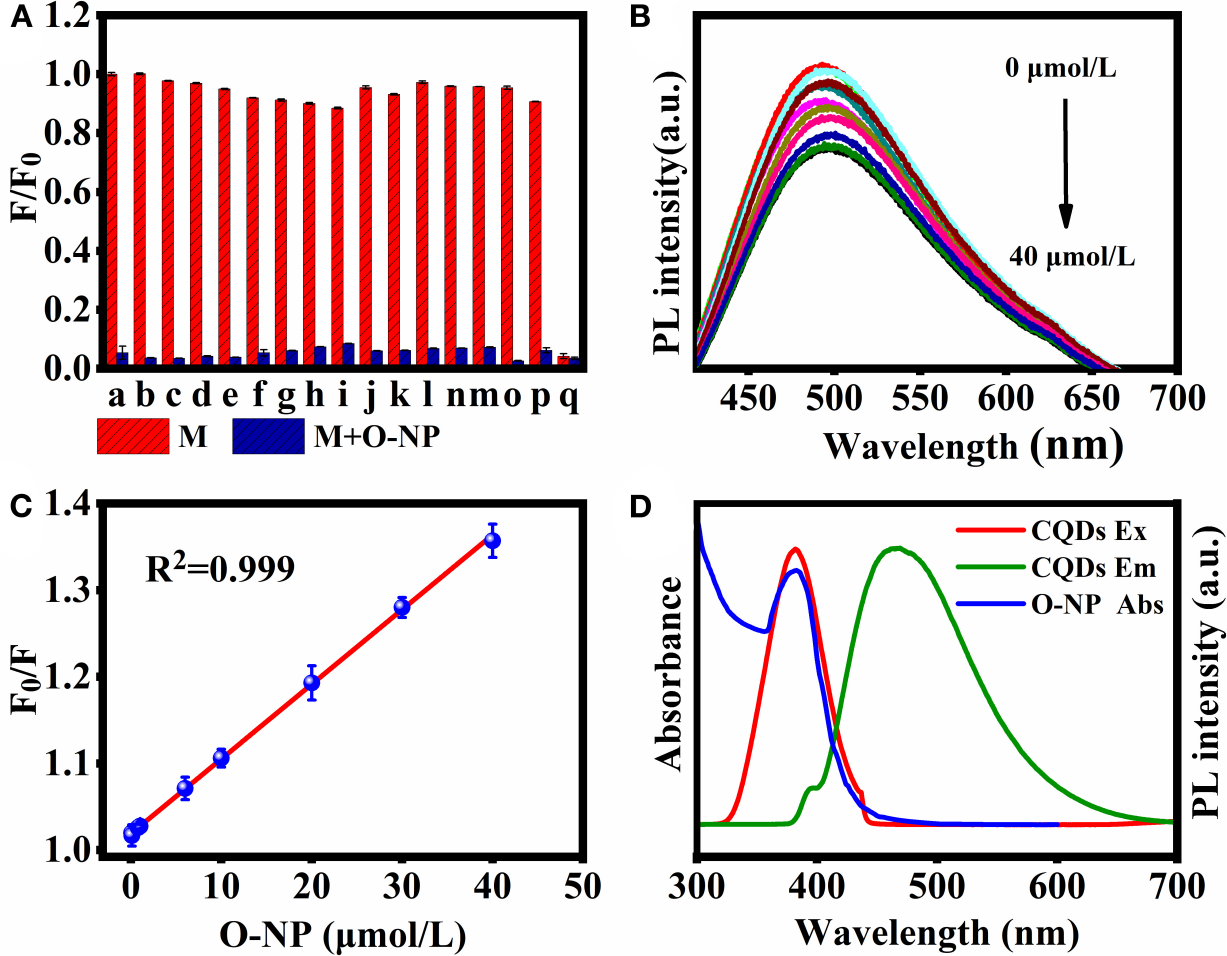
**(A)** Luminescence quenching of CQDs by single compound M (100 μmol/L, red) and in mixed solutions of M with 100 μmol/L O-NP (blue). a, blank; b, K^+^; c, Pb^2+^; d, Ba^2+^; e, Ca^2+^; f, Hg^2+^; g, Na^+^; h, Zn^2+^; i, Mg^2+^; j, Cr^3+^; k, Cu^2+^; l, 3-NT; m, 3-NP; n, DNT; o, 2,4,6-TCP; p, NB; q, O-NP. **(B)** Fluorescence spectra of CQDs in the presence of O-NP at 0–40 μmol/L (excitation: 360 nm). **(C)** Dependence of F_0_/F on the concentration of O-NP. **(D)** The absorption spectrum of O-NP, PL excitation, and emission spectra of CQDs.

Fluorescence quenching of the CQDs was measured in PBS (pH = 7.4) in the presence of K^+^, Pb^2+^, Ba^2+^, Ca^2+^, Hg^2+^, Na^+^, Zn^2+^, Mg^2+^, Cr^3+^, Cu^2+^, 3-NT, 3-NP, DNT, 2,4,6-TCP, NB, and O-NP. In Figure 5A, O-NP shows the strongest fluorescence quenching among the 16 substances, implying that these CQDs can be used as a nano sensing platform for O-NP detection. The fluorescence intensity (excitation: 360 nm) gradually decreases with increasing O-NP concentration from 0 to 40 μmol/L, confirming the system's sensitivity toward O-NP (Figure 5B). The fluorescence quenching data follow the Stern-Volmer equation (Hou et al., 2015):

$$\begin{array}{l} {\text{F}}_{0}/\text{F}=0.00862\text{C}+1.01905 \end{array}$$

where F and F_0_ are the fluorescence intensities at 360 nm in solutions with and without O-NP, respectively. C is the concentration of O-NP. There is a good linear relationship between F_0_/F and C in the range of C = 0.08–40 μmol/L, with a correlation coefficient (*R*^2^) of 0.999 and a limit of detection (S/N = 3, *n* = 3) of 15.2 nmol/L (Figure 5C). These results clearly confirmed that the CQDs can be used as a nanoprobe for O-NP detection.

### Possible Mechanism of CQD-Based Fluorescent Probes for O-NP Detection

Inner filter effect (IFE) is an important and dominant factor in fluorescence quenching measurements, which was caused by the absorption of the excitation and/or emission light by the analyte in the detection system (Yuan and Walt, 1987). One of the most important conditions for meeting IFE is that the absorption spectrum of the analyte should have a sufficient overlap region with the excitation and/or emission spectrum of the fluorescent agent (Chen et al., 2017). In order to better understand the fluorescence quenching mechanism between O-NP and CQDs, the UV-Vis absorption response of O-NP was further analyzed. As shown in Figure 5D, the UV-vis absorption spectrum of O-NP was overlaps with the excitation of the CQDs. which was meet the important conditions of IFE. Therefore, IFE was considered as a possible quenching mechanism in this fluorescence quenching process.

### Application of the Carbon Quantum Dots

#### Detecting o-nitrophenol in Water Samples

River water and sewage water samples were tested using the CQDs as fluorescent probes, showing O-NP concentration of 1.51 and 5.12 μmol/L, respectively (Table 1). To further verify the reliability of this method, the river water was spiked with O-NP standards at two different concentrations of 2.0 and 10 μmol/L. The recoveries were 94.0–110% with a relative standard deviation (RSD) of 1.4–3.6%. When the sewage water sample was spiked with O-NP standards at 5.0 and 10 μmol/L, the recoveries were 98.2–108% with RSD = 1.5–2.4%. These results demonstrated the potential of CQD-based fluorescent probes for detecting O-NP in aqueous samples.

**Table 1:** Recovery test and precision for the analysis of o-nitrophenol in water samples (*n* = 3).

**Sample**	**Original amount (μmol//L)**	**RSD (%)**	**Added (μmol/L)**	**Found (μmol/L)**	**Recovery (%)**	**RSD (%)**
River water	1.51	4.5	2.00	3.71	110	3.6
			10.0	10.9	94.0	1.4
Sewage	5.12	2.8	5.00	10.0	98.2	2.4
			10.0	15.9	108	1.5

#### Bioimaging Study

**Figure 6:**
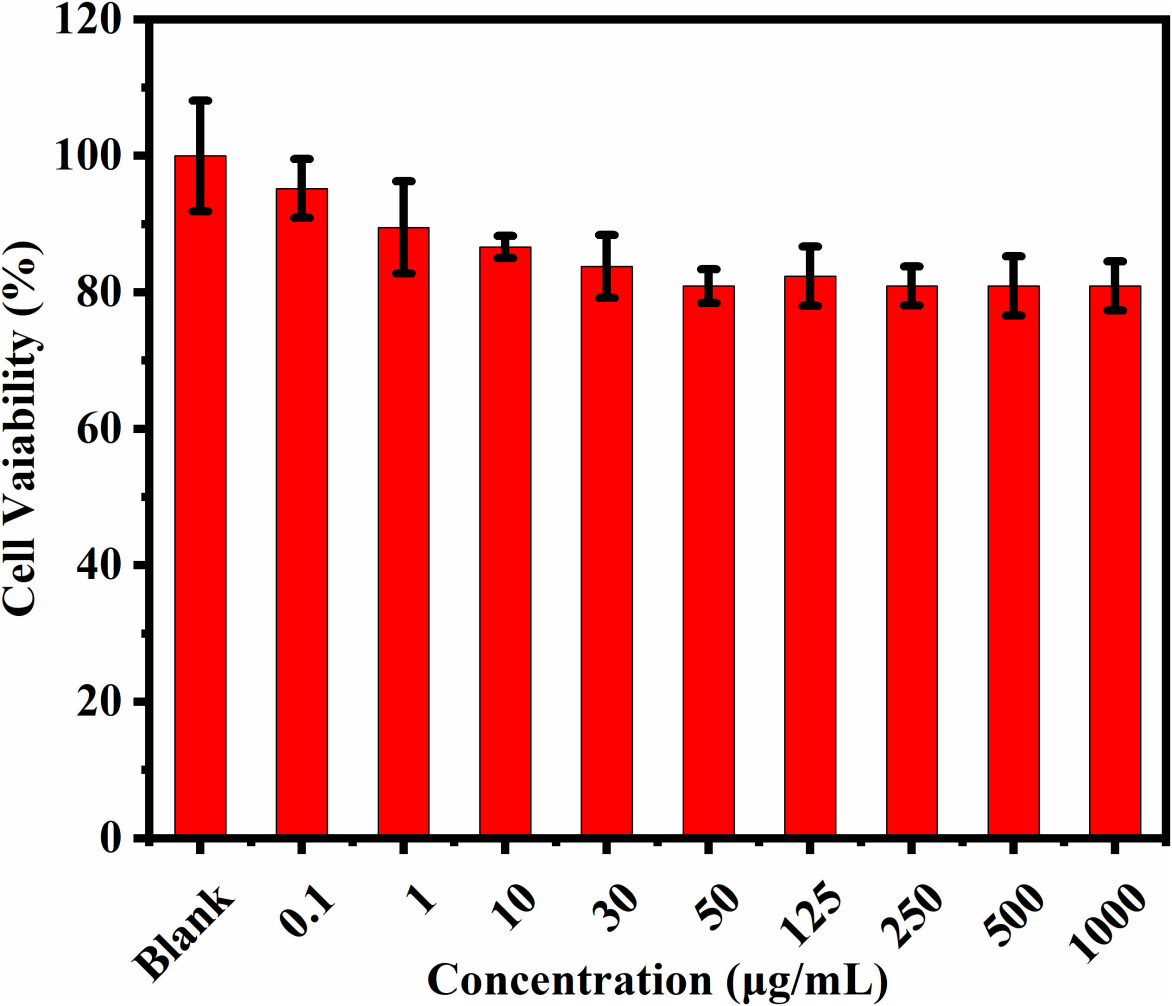
Cell viability after treatment with various concentrations of CQDs (*n* = 3).

**Figure 7:**
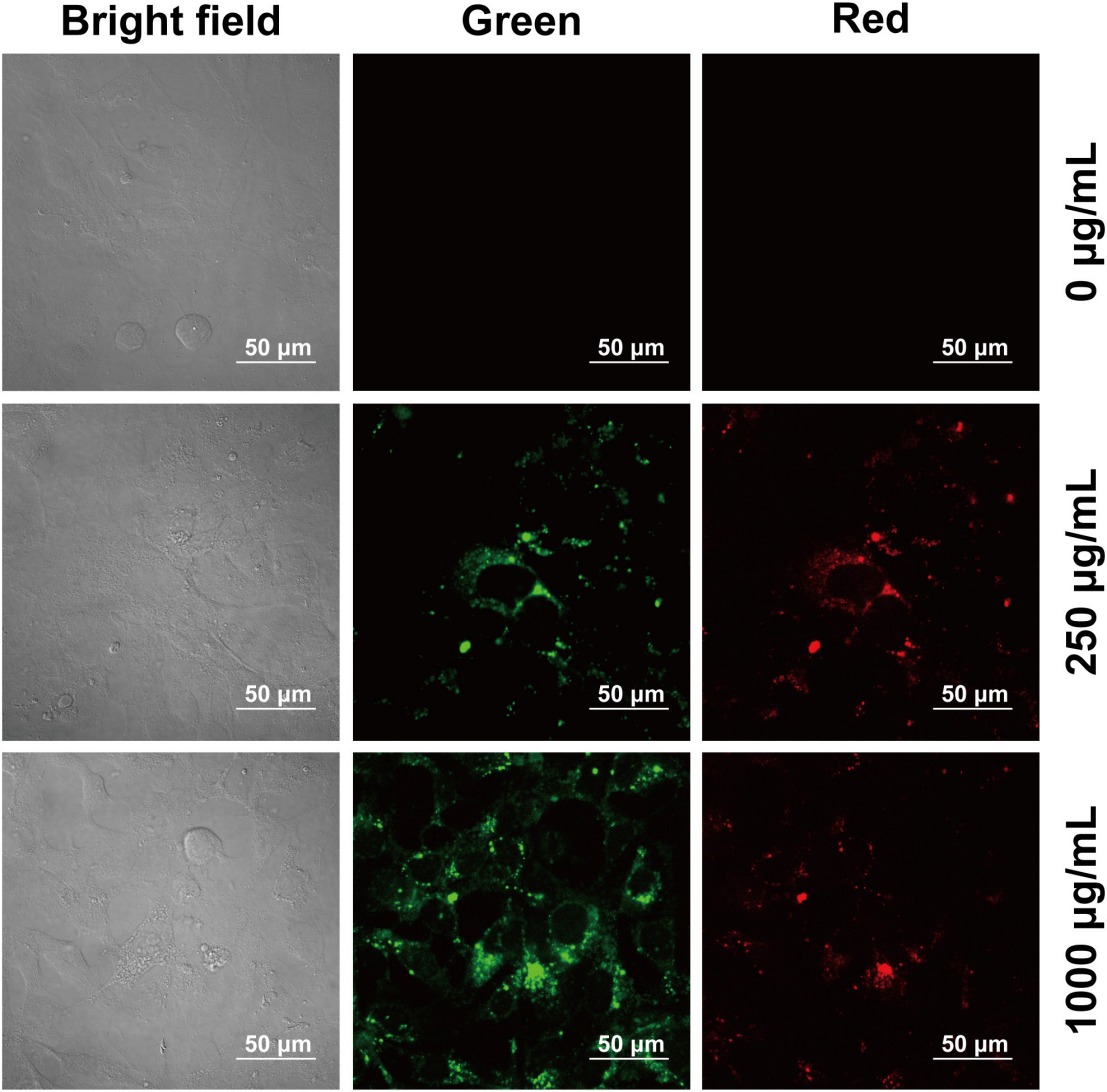
Confocal microscopy images of Hep3B cells at different excitation wavelengths after incubation with CQDs at various concentrations.

Cytotoxicity of the prepared CQDs toward Hep3B cells was examined using a standard MTT assay (Figure 6). After incubation with CQDs (0–1,000 μg/mL) for 24 h, the cell survival rate was estimated to be more than 80%. This fully confirms the low cytotoxicity and excellent biocompatibility of CQDs as a probe. Subsequently, we used a nanometer plasma confocal laser scanning microscope to investigate the distribution of CQDs after incubation to further prove their suitability for *in vitro* imaging. As shown in Figure 7, at all concentrations, the CQDs incubated with cells gave green fluorescence under 488 nm irradiation (emission wavelength: 448–508 nm) and red fluorescence under 561 nm (emission wavelength: 498–588 nm). The images in green fluorescence are clearer and more detailed than those in red fluorescence. Thus, the prepared CQDs have good biocompatibility, low cytotoxicity, and significant performance for imaging Hep3B cells *in vitro*.

## Conclusions

In summary, we report a simple, green, and economical way to synthesize carbon quantum dots using *R. roxburghii*, a widely available material, as a biomass precursor without any further passivation or modification. The hydrothermally prepared carbon quantum dots have a fluorescence yield of about 24.8% and an average diameter of about 2.5 nm with a narrow distribution. Remarkably, they exhibited good selectivity and high sensitivity to o-nitrophenol in aqueous solution, and the detection results were satisfactory. At the same time, these carbon quantum dots also showed good performance in fluorescent bioimaging, characterized by good photoluminescence effect, low cytotoxicity, and high biocompatibility. Therefore, this new carbon nanomaterial could be successfully used for *in vitro* bioimaging.

## Data Availability Statement

The datasets generated for this study are available on request to the corresponding author.

## Author Contributions

QZ and LJ contributed to the research of the experiment, revised the manuscript, and finalized the final version. JL conceived experiments for data analysis and wrote a dissertation. LZ assisted in writing and collected experimental data. YWa contributed to data analysis and collation. YWu and YZ correctly guided the use of medicines, materials, and analytical tools. All authors contributed to the article and approved the submitted version.

## Conflict of Interest

The authors declare that the research was conducted in the absence of any commercial or financial relationships that could be construed as a potential conflict of interest.
