# Supplementary figures and images for: Synthesis of Novel Fluorescent Carbon Quantum Dots From Rosa roxburghii for Rapid and Highly Selective Detection of o-nitrophenol and Cellular Imaging
Source: Front Chem. 2020 Jul 31;8:665. doi: 10.3389/fchem.2020.00665 (PMC7411352; doi:10.3389/fchem.2020.00665)

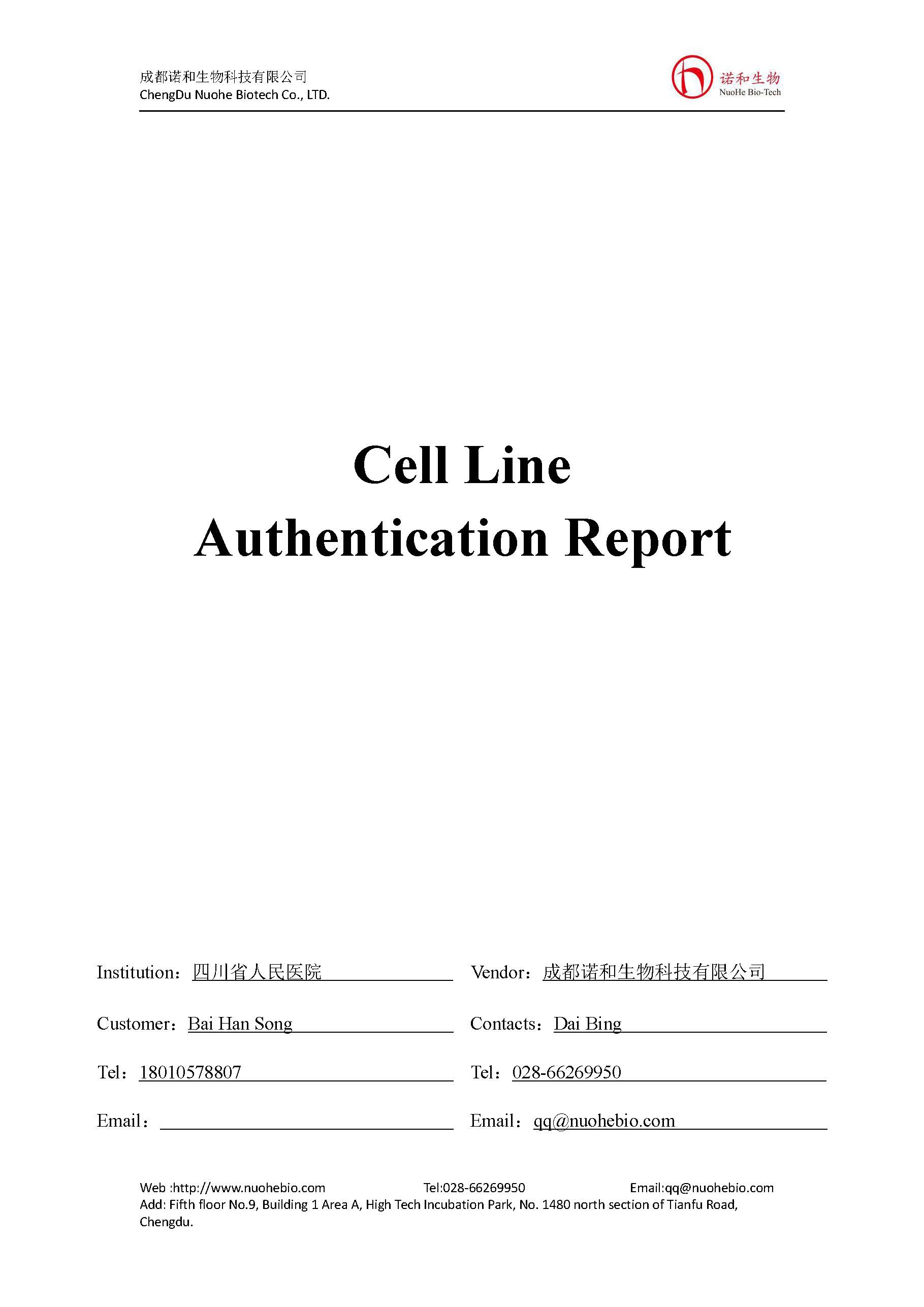

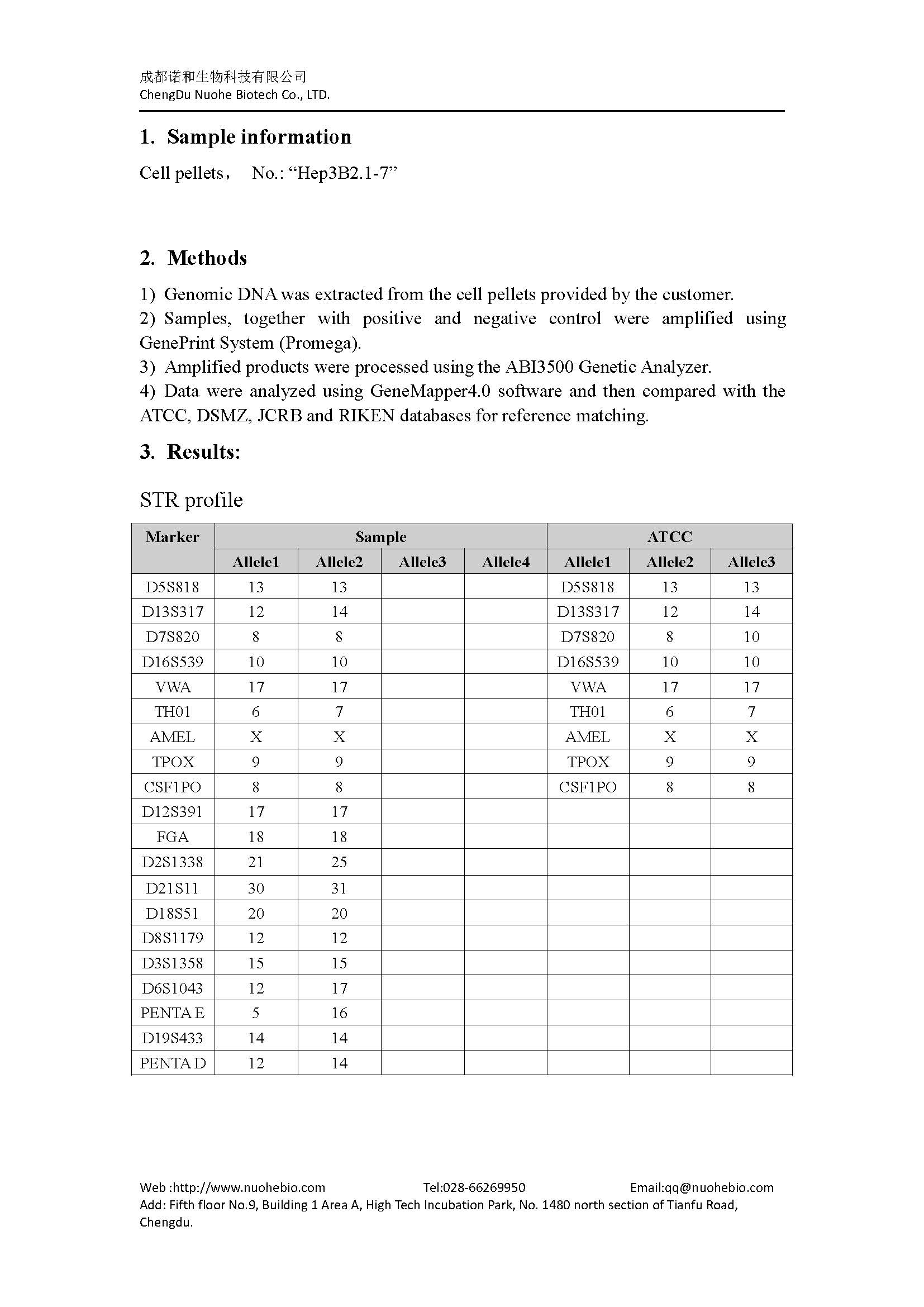

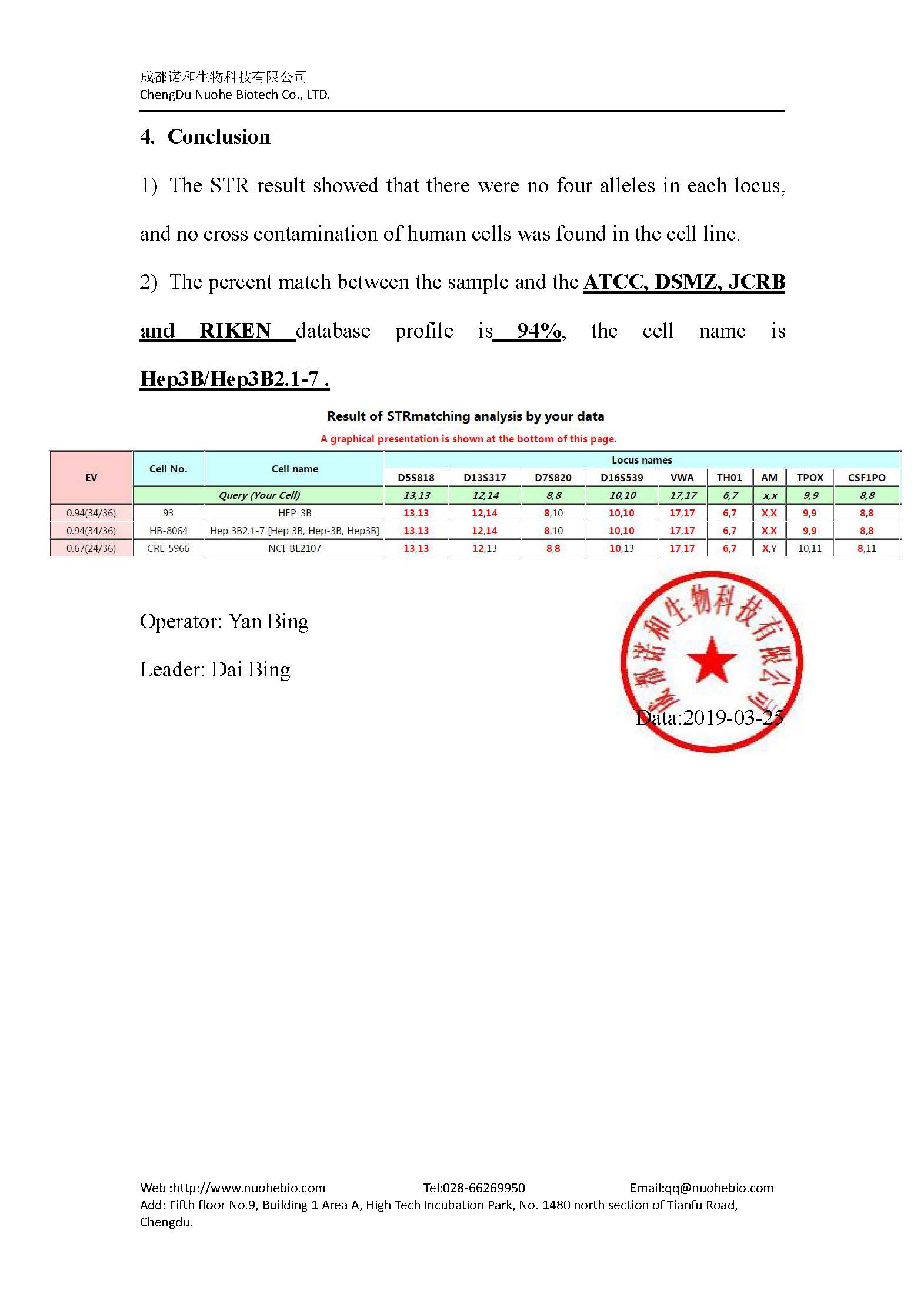

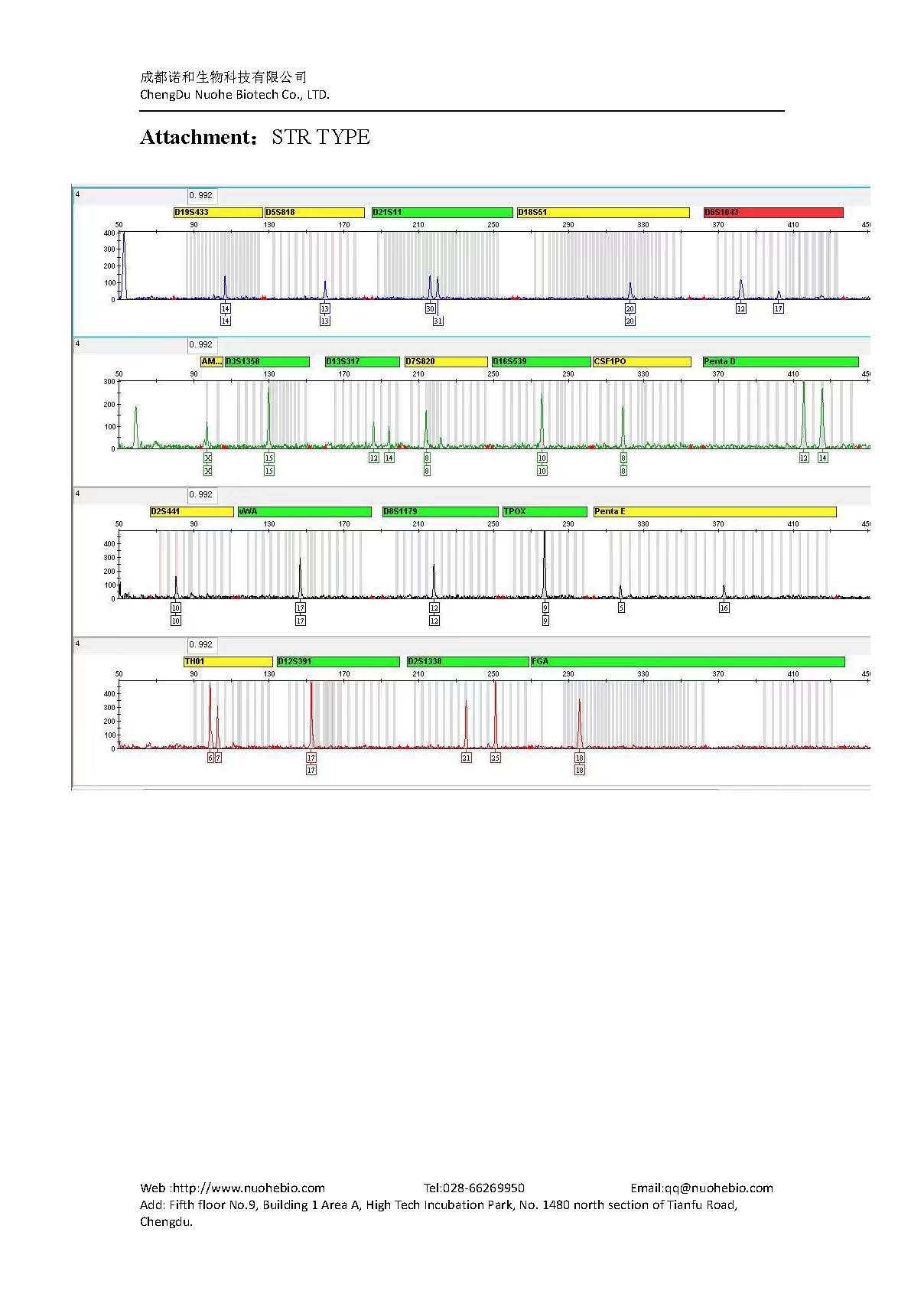

Supplement: Supplementary file 1 [file Table_1.DOCX]
